# Prognosis of patients with operated chronic subdural hematoma

**DOI:** 10.1038/s41598-022-10992-5

**Published:** 2022-04-29

**Authors:** Jussi P. Posti, Teemu M. Luoto, Jussi O. T. Sipilä, Päivi Rautava, Ville Kytö

**Affiliations:** 1 Neurocenter, Department of Neurosurgery and Turku Brain Injury Center, Turku University Hospital and University of Turku, P.O. Box 52, 20521 Turku, Finland; 2 Department of Neurosurgery, Tampere University Hospital and Tampere University, Tampere, Finland; 3 Clinical Neurosciences, University of Turku, Turku, Finland; 4 Department of Neurology, Siun Sote, North Karelia Central Hospital, Joensuu, Finland; 5 Clinical Research Center, Turku University Hospital and University of Turku, Turku, Finland; 6 Heart Centre and Center for Population Health Research, Turku University Hospital and University of Turku, Turku, Finland; 7 Research Center of Applied and Preventive Cardiovascular Medicine, University of Turku, Turku, Finland; 8 Center for Population Health Research, Turku University Hospital and University of Turku, Turku, Finland; 9 Administative Center, Hospital District of Southwest Finland, Turku, Finland

**Keywords:** Neurology, Prognosis

## Abstract

Chronic subdural hematoma (cSDH), previously considered fairly benign and easy to treat, is now viewed a possible sign of incipient clinical decline. We investigated case-fatality, excess fatality and need for reoperations following operated cSDH in a nationwide setting focusing on patient-related characteristics. Finnish nationwide databases were searched for all admissions with operated cSDH as well as later deaths in adults (≥ 16 years) during 2004–2017. There were 8539 patients with an evacuated cSDH (68% men) with a mean age of 73.0 (± 12.8) years. During the follow-up, 3805 (45%) patients died. In-hospital case-fatality was 0.7% (n = 60) and 30-day case-fatality 4.2% (n = 358). The 1-year case-fatality was 14.3% (95% CI = 13.4–15.2%) among men and 15.3% (95% CI = 14.0–16.7%) among women. Comorbidity burden, older age, and alcoholism were significantly associated with fatality. One-year excess fatality rate compared to general Finnish population was 9.1% (95% CI = 8.4–9.9) among men and 10.3% (95% CI = 9.1–11.4) among women. Highest excess fatality was observed in the oldest age group in both genders. Reoperation was needed in 19.4% (n = 1588) of patients. Older age but not comorbidity burden or other patient-related characteristics were associated with increased risk for reoperation. The overall case-fatality and need for reoperations declined during the study era. Comorbidities should be considered when care and follow-up are planned in patients with cSDH. Our findings underpin the perception that the disease is more dangerous than previously thought and causes mortality in all exposed age groups: even a minor burden of comorbidities can be fatal in the post-operative period.

## Introduction

Chronic subdural hematoma (cSDH) is the most common type of intracranial hemorrhage among older people^1^ and has become one of the most common neurosurgical diseases in the Western World due to the ageing population structure^2,3^. It is further anticipated that improved access to head imaging and more frequent use of antithrombotic medication will result in continuing increase in the incidence of cSDH^4^. Paradoxically, there are only a few large-scale studies of a disease of such public health significance^5,6^.

In current practice, asymptomatic patients with cSDH are managed with conservative measures including anticoagulation reversal/pausing and serial head imaging. For symptomatic patients with acceptable surgical risk, the treatment of choice is a burr-hole craniostomy with irrigation followed by a subdural drainage^7–9^. The surgical procedure is mini-invasive and is usually performed under local anesthesia^7^ and is therefore considered a minor intervention.

Mortality after operated cSDH has generally been associated with high average age and frailty^3,10,11^. Recently, however, perceptions of the disease have changed, especially as high one-year case-fatality and excess-fatality rates in all treated age groups have been reported^6,12,13^. Coined as a sentinel health event^14^, cSDH may be the beginning of a deterioration in health and may exacerbate or reveal previous asymptomatic diseases^15,16^.

Recent results from a large study including consecutive cSDH patients from Pirkanmaa region, Finland, show that patients with cSDH of all ages have continuous excess fatality up to 20 years after diagnosis. Patient-related characteristics exhibit a strong association with excess mortality, while cSDH-related pathophysiological findings do not^6^. We therefore designed a study to examine the aftermath of operated cSDH in a nationwide registry setting focusing on patient-related characteristics in different age groups.

## Materials and methods

### Patients and study design

For this study, all patients aged ≥ 16 years with neurosurgical or intensive care ward admission for traumatic and non-traumatic cSDH [International Classification of Diseases, 10th revision (ICD-10) diagnosis codes S06.5 or I62.0 as any diagnosis] and evacuation of subdural hematoma (Nordic Medico-Statistical Committee, NOMESCO codes AAD10 and/or AAD12^17^) from January 1, 2004, to December 31, 2017 were identified from the Care Register for Health Care. The year 2018 was included as a follow-up period without including new patients. This mandatory-by-law database includes all public health care hospital admissions in Finland. Neurosurgical services are provided by all five university hospitals in Finland (Helsinki, Tampere, Turku, Kuopio and Oulu) of which all were included in the search. Only the first admission per patient was included. Patients with missing survival follow-up data (n = 19) were excluded.

The validity of the study population was assessed by reviewing patient records of 200 randomly selected neurosurgical patients admitted to Turku University Hospital with an ICD-10 traumatic brain injury code S06.* (in which cSDH is included) as the primary discharge diagnosis . Of reviewed patients, 198 fulfilled the diagnostic criteria for S06.* resulting in a positive predictive value of 0.99.

Outcomes of interest were death by any cause and reoperation (AAD10 or AAD12) within 1-year and 10-years. Follow-up ended on December 31, 2018, or upon death, whichever came first. Median follow-up of survivors was 5.2 year (range 1–10 years).

Fatality data were obtained from Statistics Finland, the national census entity. Reoperations were detected from the Care Register for Health Care in Finland. Relevant comorbidities were identified using the ICD-10 coding. Charlson Comorbidity Index (CCI) score including AIDS/HIV, dementia, diabetes, chronic pulmonary disease, cerebrovascular disease, heart failure, hemi- or paraplegia, liver disease, malignancies, myocardial infarction, peptic ulcer disease, peripheral vascular disease, rheumatic disease, and renal disease was calculated as previously described^18^. Excess case-fatality after cSDH was calculated by subtracting the baseline fatality from the observed case-fatality. Baseline fatality was calculated using gender-, age-, and calendar year-specific expected fatality rates in the corresponding total Finnish population provided by the Statistics Finland (www.stat.fi). Admission duration was calculated as beginning days and included only the primary hospital admission in a neurosurgical center.

The study was approved by the National Institute for Health and Welfare of Finland (THL, permission no: THL/2245/5.05.00/2019) and Statistics Finland (TK-53-484-20). This was a retrospective register study, and thus ethical board review and requirement for informed consent were waived, and the participants were not contacted. The legal basis for processing personal data is public interest and scientific research (EU General Data Protection Regulation 2016/679, Article 6(1)(e) and Article 9(2)(j); Data Protection Act, Sections 4 and 6).

### Statistical analysis

Baseline features were analyzed with independent samples *t*-test or Chi-squared test as appropriate. Outcomes were analyzed with the Kaplan–Meier method and Cox-regression. Multivariable Cox models included age, gender, CCI, alcohol abuse, atrial fibrillation, coagulopathy, hypertension, and study era which all were deemed clinically relevant for modelling. All Cox-models were adjusted for surgical center by stratification. The results are given as the mean, median, percentage, hazard ratio (HR), or relative risk (RR) with a 95% confidence interval (CI), interquartile range (IQR), or ± standard deviation (SD). Statistical significance was defined as a *p* value of < 0.05. Analyses were performed with SAS version 9.4 (SAS Institute, Inc., Cary, NC, USA; https://support.sas.com/software/94/).

### Ethical approval and informed consent

This is a retrospective registry study and no approval from an ethical committee was required.

## Results

During the study years 2004–2017, there were 8539 patients with evacuated cSDH (68% men). Women were older than men, among whom alcohol abuse and hypertension were more common (Table 1).

**Table 1:** Baseline characteristics of the study patients.

Variable	Total	Women	Men	*p* value*
	n = 8539	n = 2724	n = 5815	
Age, mean (SD)	73.0 (12.8)	75.6 (12.4)	71.8 (12.8)	< 0.0001
16–54	735 (8.6%)	172 (6.3%)	563 (9.7%)	
55–64	1216 (14.2%)	321 (11.8%)	895 (15.4%)	
65–74	2097 (24.6%)	553 (20.3%)	1544 (26.6%)	
75–84	2971 (34.8%)	989 (36.3%)	1982 (34.1%)	
≥ 85	1520 (17.8%)	689 (25.3%)	831 (14.3%)	
**CCI**				< 0.0001
0	3447 (40.4%)	1031 (37.9%)	2416 (41.2%)	
1	2185 (25.6%)	785 (28.8%)	1400 (24.1%)	
2	1405 (16.5%)	448 (16.5%)	957 (16.5%)	
3	774 (9.1%)	252 (9.3%)	522 (9.0%)	
≥ 4	728 (8.5%)	208 (7.6%)	520 (8.9%)	
Alcohol abuse	799 (9.4%)	145 (5.3%)	654 (11.3%)	< 0.0001
Atrial fibrillation	1892 (22.2%)	603 (22.1%)	1289 (22.2%)	0.975
Coagulopathy	89 (1.0%)	25 (0.9%)	64 (1.1%)	0.438
Hypertension	2746 (32.2%)	1006 (36.9%)	1740 (29.9%)	< 0.0001
**Study era**				0.552
2004–2008	2461 (28.8%)	764 (28.1%)	1697 (29.2%)	
2009–2013	3099 (36.3%)	1002 (36.8%)	2097 (36.1%)	
2014–2017	2979 (34.9%)	958 (35.2%)	2021 (34.8%)	

*p-value between the genders.

Of the patients, 3805 died during the 10-year follow-up. The in-hospital case-fatality rate was 0.7% (n = 60) and 30-day fatality rate 4.2% (n = 358). The median admission duration after surgery was three days with an interquartile range of 2–4 days.

In both genders, the highest case-fatality rates were observed in the oldest age group (Table 2). In the 1-year case-fatality multivariable model, age groups of 75 years or older, CCI score 1 or above, atrial fibrillation, alcohol abuse increased HR for death. Higher CCI and older age had the highest HRs for case-fatality (Table 3 and Supplementary Figure S1). The two latest study eras (2009–2013 and 2014–2018) were associated with decreased HR for case-fatality when the first study era (2004–2008) was used as a reference (Table 3).

**Table 2:** One-year absolute and excess case-fatality of operated cSDH patients and relative risk of death compared to corresponding general population.

Men (years)	Patients	Baseline-fatality % (95% CI)	cSDH case-fatality % (95% CI)	Excess fatality % (95% CI)	RR (95% CI)
16–54	563	0.4 (0.0–0.9)	8.7 (6.4–11.0)	8.3 (6.0–10.6)	22.9 (5.9–89.7)
55–64	895	1.1 (0.4–1.8)	10.2 (8.2–12.1)	9.1 (7.2–1.0)	9.3 (4.8–17.9)
65–74	1544	2.4 (1.6–3.1)	8.7 (7.3–10.2)	6.4 (5.2–7.6)	3.7 (2.6–5.3)
75–84	1982	6.1 (5.0–7.1)	15.4 (13.8–17.0)	9.3 (8.1–10.6)	2.5 (2.1–3.1)
≥ 85	831	15.9 (13.4–18.3)	30.1 (27.0–33.2)	14.2 (11.8–16.6)	1.9 (1.6–2.3)
Total	5815	5.2 (4.6–5.7)	14.3 (13.4–15.2)	9.1 (8.4–9.9)	2.8 (2.4–3.1)

Women (years)	Patients	Baseline-fatality % (95% CI)	cSDH case-fatality % (95% CI)	Excess fatality % (95% CI)	RR (95% CI)
16–54	172	0.2 (0.0–0.8)	3.5 (0.7–6.2)	3.3 (0.6–6.0)	20.1 (0.5–783.5)
55–64	321	0.5 (0.0–1.3)	10.0 (6.7–13.2)	9.5 (6.3–12.7)	20.4 (4.1–100.6)
65–74	553	1.2 (0.3–2.0)	8.7 (6.3–11.0)	7.5 (5.3–9.7)	7.5 (3.3–17.1)
75–84	989	4.0 (2.8–5.2)	14.1 (11.9–16.2)	10.0 (8.2–11.9)	3.5 (2.5–4.9)
≥ 85	689	12.9 (10.4–15.4)	27.9 (24.5–31.2)	15.0 (12.3–17.6)	2.2 (1.7–2.7)
Total	2724	5.0 (4.2–5.8)	15.3 (14.0–16.7)	10.3 (9.1–11.4)	3.0 (2.5–3.7)

95% Confidence intervals are presented in the parentheses. cSDH, chronic subdural hemorrhage. RR, Relative Risk (cSDH vs. general population).

**Table 3:** Features associated with 1–year case–fatality after index surgery for cSDH. Results of univariable and multivariable analyses.

Variable	1-year case-fatality				
	Rate	Univariable		Multivariable	
		HR (95% CI)	p-value	HR (95% CI)	p-value
**Gender**					
Women	15.3%	Reference		Reference	
Men	14.3%	0.92 (0.82–1.04)	0.176	1.04 (0.92–1.17)	0.546
**Age, years**			< 0.0001		< 0.0001
16–54	7.5%	Reference		Reference	
55–64	10.1%	1.36 (0.99–1.88)	0.056	1.27 (0.92–1.75)	0.141
65–74	8.7%	1.17 (0.86–1.58)	0.318	1.01 (0.74–1.38)	0.956
75–84	15.0%	2.06 (1.56–2.73)	< 0.0001	1.62 (1.21–2.18)	< 0.0001
≥ 85	29.1%	4.35 (3.29–5.76)	< 0.0001	3.24 (2.40–4.39)	< 0.0001
**CCI**			< 0.0001		< 0.0001
0	7.1%	Reference		Reference	
1	13.6%	1.98 (1.68–2.35)	< 0.0001	1.79 (1.50–2.12)	< 0.0001
2	19.1%	2.87 (2.41–3.42)	< 0.0001	2.46 (2.05–2.95)	< 0.0001
3	25.2%	3.92 (3.25–4.73)	< 0.0001	3.21 (2.64–3.91)	< 0.0001
≥ 4	33.7%	5.62 (4.71–6.71)	< 0.0001	4.61 (3.80–5.59)	< 0.0001
**Alcohol abuse**					
No	14.7%	Reference		Reference	
Yes	14.3%	0.97 (0.80–1.18)	0.748	1.39 (1.13–1.71)	0.002
**Atrial fibrillation**					
No	12.1%	Reference		Reference	
Yes	23.6%	2.07 (1.85–2.33)	< 0.0001	1.36 (1.20–1.54)	< 0.0001
**Coagulopathy**					
No	14.5%	Reference		Reference	
Yes	22.5%	1.65 (1.06–2.57)	0.026	1.38 (0.88–2.15)	0.159
**Hypertension**					
No	13.0%	Reference		Reference	
Yes	18.0%	1.41 (1.26–1.58)	< 0.0001	0.93 (0.82–1.05)	0.223
**Study era**			0.542		< 0.0001
2004–2008	13.9%	Reference		Reference	
2009–2013	15.1%	1.08 (0.94–1.24)	0.271	0.83 (0.72–0.96)	0.019
2014–2017	14.6%	1.04 (0.90–1.20)	0.593	0.69 (0.60–0.81)	< 0.0001

CCI, Charlson co–morbidity index score; HR, hazard ratio; CI, confidence interval.

**Figure 1:**
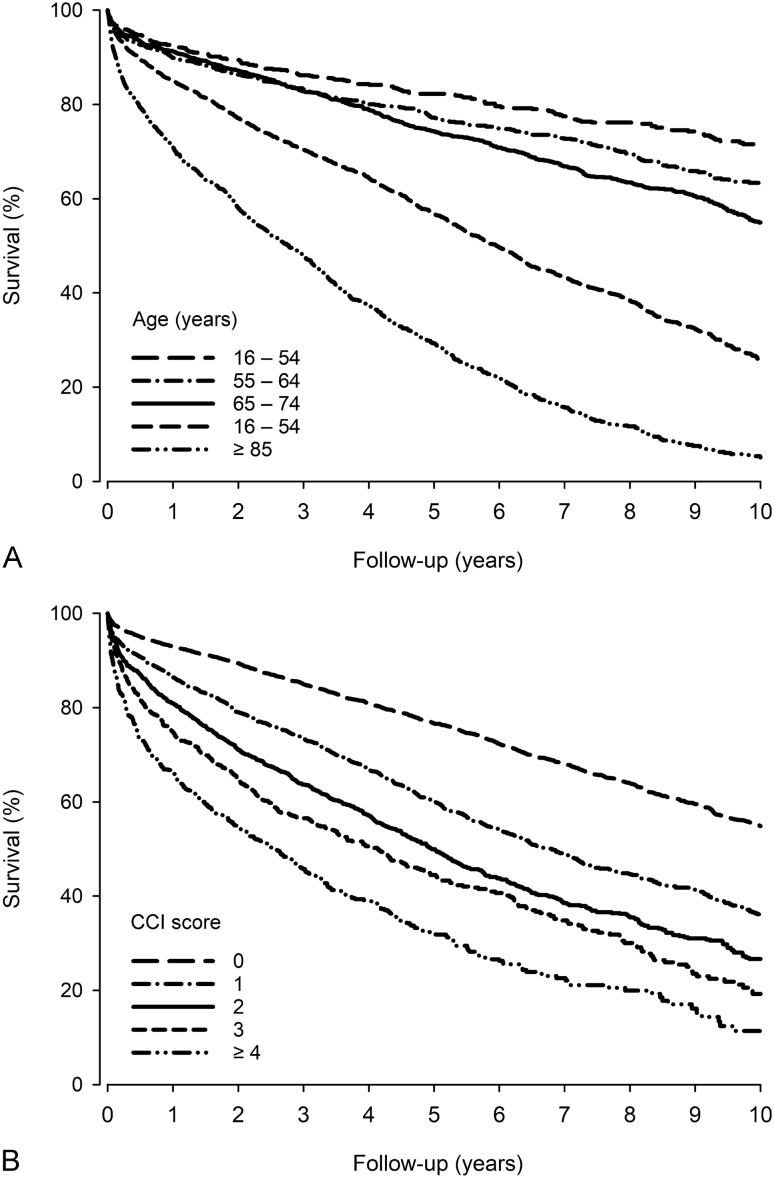
Survival after chronic subdural hematoma evacuation by age (**A**) and Charlson comorbidity index (CCI) score (**B**).

Ten-year case-fatality rate was 60.2%. In the multivariable analysis, increased HR for 10-year case fatality was associated with the age groups of 55 years or older (with the youngest age group as a reference), CCI score 1 or above (with CCI score of 0 as a reference), alcohol abuse and atrial fibrillation (Supplementary Table S1 and Supplementary Figure S2). Again, higher CCI and older age had the highest HRs for case-fatality, which is also demonstrated in Fig. 1.

Highest one-year excess fatality rates compared to the general population were observed in the oldest age group followed by age groups of 75–84 years and 55–64 years in both genders. Highest RR for death was observed in the age group of 16–54 years in men and 55–64 years in women (Table 2). For this study, reference population is the whole corresponding Finnish background population at the time of the study, 2014–2018 (n = 61,962,815 person-years).

Reoperation was performed on 1588 patients yielding a reoperation rate of 19.4% within one year. Only one patient was reoperated later: on the 368th postoperative day. In the 1-year reoperation multivariable model, all older age groups (with the youngest age group as a reference) were associated with increased HR for reoperation (Table 4 and Supplementary Figure S3). The latest study era (with the first study era as a reference) was associated with decreased HR for reoperation (Table 4).

**Table 4:** Features associated with re–operation within one year from index operation for cSDH.

Variable	Reoperation within 1 year				
	Rate	Univariable		Multivariable	
		HR (95% CI)	*p*-value	HR (95% CI)	*p*-value
**Gender**					
Women	14.0%	Reference		Reference	
Men	21.9%	1.61 (1.44–1.81)	< 0.0001	1.68 (1.49–1.89)	< 0.0001
**Age, years**			< 0.0001		< 0.0001
16–54	12.3%	Reference		Reference	
55–64	16.3%	1.35 (1.05–1.74)	0.020	1.38 (1.07–1.77)	0.014
65–74	19.8%	1.64 (1.30–2.07)	< 0.0001	1.67 (1.32–2.11)	< 0.0001
75–84	22.2%	1.87 (1.50–2.34)	< 0.0001	1.95 (1.54–2.46)	< 0.0001
≥ 85	19.6%	1.61 (1.27–2.05)	0.0001	1.78 (1.38–2.30)	< 0.0001
**CCI**			0.749		0.956
0	19.1%	Reference		Reference	
1	19.0%	0.98 (0.86–1.11)	0.733	0.98 (0.86–1.12)	0.765
2	20.8%	1.08 (0.94–1.25)	0.264	1.04 (0.90–1.21)	0.575
3	20.0%	1.04 (0.87–1.24)	0.695	1.01 (0.83–1.21)	0.957
≥ 4	19.4%	1.02 (0.84–1.23)	0.875	0.99 (0.81–1.21)	0.892
**Alcohol abuse**					
No	19.9%	Reference		Reference	
Yes	14.8%	0.73 (0.60–0.88)	0.001	0.82 (0.67–1.00)	0.051
**Atrial fibrillation**					
No	19.2%	Reference		Reference	
Yes	20.4%	1.07 (0.95–1.20)	0.274	1.01 (0.89–1.15)	0.852
**Coagulopathy**					
No	19.5%	Reference		Reference	
Yes	16.2%	0.80 (0.46–1.38)	0.414	0.83 (0.48–1.44)	0.512
**Hypertension**					
No	19.7%	Reference		Reference	
Yes	18.9%	0.93 (0.83–1.03)	0.155	0.92 (0.82–1.03)	0.138
**Study era**			0.143		0.066
2004–2008	20.3%	Reference		Reference	
2009–2013	19.5%	0.94 (0.84–1.06)	0.942	0.94 (0.83–1.06)	0.293
2014–2017	18.6%	0.88 (0.78–1.00)	0.049	0.86 (0.76–0.98)	0.021

Results of univaribale and multivariable analyses.

CCI, Charlson co–morbidity index score; HR, hazard ratio; CI, confidence interval.

## Discussion

The main findings of this nationwide study are that after operated cSDH (i) the 1-year case-fatality was about 15%––the highest case-fatality rates were observed in the oldest age group, (ii) comorbidities drastically increase fatality, (iii) one-year excess fatality rate compared to the general population was about 10%––the highest risk for fatality was observed among the youngest age group, (iv) older age but not comorbidities increase the risk for reoperations, and (v) case-fatality and the need for reoperations is declining in Finland over time.

cSDH was long considered a condition that is trivial, benign in nature and easy to treat, most likely because of the straightforwardness of its surgical procedure^15,19^. During the last two decades, however, perception of the nature of the disease has changed: cSDH has been associated with higher lingering mortality rates than previously reported^6,12,14,15^. Alcohol abuse resulting in the triad of brain atrophy, coagulation dysfunction, and risk for incidental falls^9,20^, antithrombotic treatment^21^, and older age^7^ are the most well-known risk factors for cSDH. Lately, the condition has been regarded as an event, which often leads to decline in health in older individuals, although not necessarily being a direct cause for decline but an indicator of subsequent deterioration^14^ or an aggravating factor for other underlying diseases^15^. Frailty has been associated with worse outcomes in other neurosurgical conditions^22,23^, and more recently, also in patients with operated cSDH^11^. Moreover, because operated cSDH is associated with excess fatality in all affected age groups in Finland^6^, then main aim of the current study was to examine the association of patient-related characteristics––comorbidities in focus––with case- and excess fatality and the need for reoperations in a nationwide setting in Finland.

In-hospital and 1-year mortality rates after operated cSDH vary across studies. The in-hospital mortality rate of 0.7% in the current Finnish nationwide study is low compared to earlier reports of rates as high as 8–19%^14,15^. The current 30-day mortality rate of 4.2% is similar compared to the report by Rauhala and colleagues^6^. It is noteworthy that the patient cohort of the Rauhala et al. study is partly included in the nationwide cohort of the current study. Our 1-year case-fatality rate for men was 14% and 15% for women. Our findings are similar to the recent regional Finnish study^6^ and another nationwide Finnish study examining the association of dementia and mortality after operated cSDH^24^, but significantly lower than previously reported by Dumont et al. (30%)^14^ and Miranda et al. (32%)^15^. The current results indicate that the case-fatality rate after operated cSDH is temporally decreasing in Finland. Some reasons for the decrease in mortality can be presented. The procedure is now performed almost invariably under local anesthesia, and the avoidance of general anesthesia. Shorter duration of surgery has possibly contributed to a decrease in mortality, especially in older patients. Whitehouse et al. reported that anesthesia duration was a risk factor for one-year mortality in a cohort predominantly consisting of patients with cSDHs^25^. We have recently reported that the number of acute trauma craniotomies and later mortality are decreasing in Finland^26^. Epidemiological changes in acute traumatic brain injuries may also affect patients with cSDH on a larger scale, although this condition does not typically manifest in the acute phase.

We observed 1-year excess case-fatality in all age groups, the total rate being 9–10%. The excess fatality was lowest in the age group of 16–54 years among women, but unexpectedly among men, in the age group of 65–75 years. Excess case-fatality rate was 8% among men and only 3% among women in the youngest age group. Intuitively, relative risks for death were high (RR 9–23) in patients younger than 65 years. In the recent regional Finnish study, excess case-fatality was also observed in all age groups with higher risk for death in patients who were treated conservatively^6^. Cumulative excess case-fatality has been also observed in other previous studies ranging from 1 to 5 years^12–15^.

Recurrence rates of both asymptomatic cSDH and cSDH requiring reoperation also widely vary across studies. Recurrence rate may be as high as 70% over time, but modern estimated reoperation rates range between 10 and 20%^3–5,7^. Our current reoperation rate of 19% barely fits in this range of reoperation rates. Of note, the majority of cSDH recurrences develop with the first two months after the operation^27^, therefore some of the 1-year reoperations included in this study can be related to a contralateral cSDH. Most likely the true cSDH recurrence rate in Finland is a bit lower than 19%. We observed that higher age, but no other patient-related characteristics, were associated with increased risk for reoperation. Alcohol abuse was associated with decreased risk for reoperation. This may reflect the clinical practice: (i) patients with chronic alcoholism often present with cortical atrophy, and the persistence of a subdural collection, if well tolerated, are not always systematically re-operated, and (ii) patients with chronic alcoholism may be more often treated conservatively after cSDH recurrence due to their poor health or lack of commitment to treatment (e.g., drains and clinical follow-up). One of the main findings is that although our reoperation rate can be considered high in the light of the current literature, the rate of reoperations is temporally decreasing in Finland. This is probably due to centralization of operations to university hospitals and standardization of drain usage.

We observed a significant association of comorbidities and case-fatality after operated cSDH, which is consistent with another recent study^11^. The results of this study show that the association of even minor comorbidity burden and mortality is evident already in the youngest age group and the association increases with age. We also observed that in both 1-year and 10-year case-fatality models, atrial fibrillation and alcohol abuse were associated with increased risk for death. It has been reported that patient-related characteristics such disability may be more important contributing factor to case-fatality after cSDH than cSDH itself and its clinical/radiological features^6^. Especially dementia––a major contributor to frailty––is shown to be an independent risk factor for 1-year case fatality after a diagnosis of cSDH^6,24^. These observations are consistent with the proposed concept of cSDH being a sentinel health event that may result in deterioration in health and aggravating previous diseases^14,15,24,28^. It can be concluded that patients with diseases causing brain atrophy, such as dementia and chronic alcoholism in particular, are at risk of health deterioration and death after a diagnosis of cSDH.

The strengths of the study are the Finnish obligatory national databases and nationwide study design. The current results complement earlier research from Finland and support the growing body of literature indicating that cSDH is not a trivial condition. This study has also limitations that should be addressed. Due to retrospective and registry-based design, we cannot draw any causative relationships, but only examine associations. ICD-10 codes were used to retrospectively identify all the included patients. There is a possibility that some patients have not been included due to inconsistent ICD-10 and NOMESCO coding at the hospitals, which results in underestimation of the actual number of cSDH cases. We included only university hospitals where surgical care of cSDH is centralized in Finland. This ensures a more complete recording of diagnoses and surgery codes, but also leads to an underestimation of the number of cases, as operations have still been undertaken in central hospitals during the earlier study years. In addition, we reviewed randomly selected patients with traumatic brain injury from one independent center and found a positive predictive value of 0.99 for brain injury diagnoses. Tommiska and colleagues recently reported that 89% of cSDH operations were performed in university hospitals in Finland during 1997–2014^24^. Due to the registry-based nature of the data collection, the observed reoperation rate can include patients treated due to a contralateral cSDH. Thus, the reoperation rates do not perfectly reflect the true cSDH recurrence rates, though the number of patients in this group (contralateral cSDH) can be considered minor. In Finland, operations have been performed using rinsing of the subdural space alone and drainage alone and a combination of these during the study years. Due to the limitations of ICD-10 coding, we could not study these methods separately. Although we examined excess fatality compared to age-, sex-, and calendar-year specific general Finnish population, we were unable to control for co-morbidities in this reference population.

Results of this study again challenge the concept of cSDH being a benign disease: cSDH can lead to death even in young individuals who have comorbidities, and higher age is significantly associated with both reoperations and mortality. Moreover, cSDH causes substantial excess fatality in all age groups. These findings imply that in patients with newly diagnosed cSDH, attention should be paid to diagnosing and treating patient-related modifiable factors, such alcoholism, and cardiovascular diseases. However, success has been already achieved in the operative treatment of this common disease during the studied 14 years: case-fatality and reoperation rates are nation-widely declining in Finland.

## Supplementary Information

Supplementary Information 1.

Supplementary Information 2.

Supplementary Information 3.

Supplementary Information 4.

Supplementary Information 5.

## Data Availability

Due to national data protection legislation, the register data used in this study cannot be shared without applying for permission to use the data with a specific study protocol and scientifically justified study questions.

## References

[1] Liu W, Bakker NA, Groen RJM (2014). Chronic subdural hematoma: A systematic review and meta-analysis of surgical procedures. J. Neurosurg..

[2] Kitya D (2018). Causes, clinical presentation, management, and outcomes of chronic subdural hematoma at Mbarara Regional Referral Hospital. Neurosurg. Focus.

[3] Feghali J, Yang W, Huang J (2020). Updates in chronic subdural hematoma: Epidemiology, etiology, pathogenesis, treatment, and outcome. World Neurosurg..

[4] Ducruet AF (2012). The surgical management of chronic subdural hematoma. Neurosurg. Rev..

[5] Yang W, Huang J (2017). Chronic subdural hematoma: Epidemiology and natural history. Neurosurg. Clin. N. Am..

[6] Rauhala M (2020). Long-term excess mortality after chronic subdural hematoma. Acta Neurochir..

[7] Kolias AG, Chari A, Santarius T, Hutchinson PJ (2014). Chronic subdural haematoma: Modern management and emerging therapies. Nat. Rev. Neurol..

[8] Ivamoto HS, Lemos HP, Atallah AN (2016). Surgical treatments for chronic subdural hematomas: A comprehensive systematic review. World Neurosurg..

[9] Mehta V, Harward SC, Sankey EW, Nayar G, Codd PJ (2018). Evidence based diagnosis and management of chronic subdural hematoma: A review of the literature. J. Clin. Neurosci..

[10] Ramachandran R, Hegde T (2007). Chronic subdural hematomas-causes of morbidity and mortality. Surg. Neurol..

[11] McIntyre MK (2020). The effect of frailty versus initial Glasgow coma score in predicting outcomes following chronic subdural hemorrhage: A preliminary analysis. Cureus.

[12] Manickam A, Marshman LAG, Johnston R (2016). Long-term survival after chronic subdural haematoma. J. Clin. Neurosci..

[13] Guilfoyle MR, Hutchinson PJA, Santarius T (2017). Improved long-term survival with subdural drains following evacuation of chronic subdural haematoma. Acta Neurochir..

[14] Dumont TM, Rughani AI, Goeckes T, Tranmer BI (2013). Chronic subdural hematoma: A sentinel health event. World Neurosurg..

[15] Miranda LB, Braxton E, Hobbs J, Quigley MR (2011). Chronic subdural hematoma in the elderly: Not a benign disease. Clinical article. J. Neurosurg..

[16] Uno M, Toi H, Hirai S (2017). Chronic subdural hematoma in elderly patients: Is this disease benign?. Neurol. Med. Chir..

[17] NOMESCO. Classification of Surgical Procedures. *Version 1.15* (2011) doi: 10.1371/journal.pone.0030934.

[18] Quan H (2005). Coding algorithms for defining comorbidities in ICD-9-CM and ICD-10 administrative data. Med. Care.

[19] Gelabert-González M, Iglesias-Pais M, García-Allut A, Martínez-Rumbo R (2005). Chronic subdural haematoma: Surgical treatment and outcome in 1000 cases. Clin. Neurol. Neurosurg..

[20] Sim YW, Min KS, Lee MS, Kim YG, Kim DH (2012). Recent changes in risk factors of chronic subdural hematoma. J. Korean Neurosurg. Soc..

[21] Gaist D (2017). Association of antithrombotic drug use with subdural hematoma risk. JAMA J. Am. Med. Assoc..

[22] Youngerman BE (2018). The modified frailty index and 30-day adverse events in oncologic neurosurgery. J. Neurooncol..

[23] McIntyre M (2020). Increasing frailty predicts worse outcomes and increased complications after angiogram-negative subarachnoid hemorrhages. World Neurosurg..

[24] Tommiska P, Korja M, Siironen J, Kaprio J, Raj R (2020). Mortality of older patients with dementia after surgery for chronic subdural hematoma: A nationwide study. Age Ageing.

[25] Whitehouse KJ, Jeyaretna DS, Enki DG, Whitfield PC (2016). Head injury in the elderly: What are the outcomes of neurosurgical care?. World Neurosurg..

[26] Posti J.P., Luoto T.M., Rautava P., Kytö V. (2021) Mortality after trauma craniotomy is decreasing in older adults: Nationwide population-based study. World Neurosurg. 152:e313–e320 (2021) 10.1016/j.wneu.2021.05.090 34082165

[27] Rauhala, M. *et al.* Chronic subdural hematoma—incidence, complications, and financial impact. *Acta Neurochirurgica* 162:2033–2043 (2020). 10.1007/s00701-020-04398-3 PMC7415035 32524244

[28] Bartek J (2017). Surgery for chronic subdural hematoma in nonagenarians: A Scandinavian population-based multicenter study. Acta Neurol. Scand..

