# Supplementary material for: Prognosis of patients with operated chronic subdural hematoma
Source: Sci Rep. 2022 Apr 29;12:7020. doi: 10.1038/s41598-022-10992-5 (PMC9054845; doi:10.1038/s41598-022-10992-5)
Supplement: Supplementary file 1 — Supplementary Information 1. [file 41598_2022_10992_MOESM1_ESM.docx]

|  | |  | | 10-year case-fatality | | |  |
| --- | --- | --- | --- | --- | --- | --- | --- |
|  | |  | Univariable | | | Multivariable | |
| Variable | | Rate | HR (95% CI) | | p-value | HR (95% CI) | p-value |
| Sex | |  |  | |  |  |  |
|  | Women | 60.5% | Reference | |  | Reference |  |
|  | Men | 60.0% | 0.95 (0.89–1.02) | | 0.134 | 1.12 (1.05–1.20) | 0.001 |
| Age, years | |  |  | | <0.0001 |  | <0.0001 |
|  | 16 – 54 | 28.4% | Reference | |  | Reference |  |
|  | 55 – 64 | 36.9% | 1.37 (1.14–1.65) | | 0.0008 | 1.30 (1.08–1.56) | <0.0001 |
|  | 65 – 74 | 45.0% | 1.66 (1.40–1.96) | | <0.0001 | 1.51 (1.27–1.80) | <0.0001 |
|  | 75 – 84 | 74.1% | 3.40 (2.90–3.99) | | <0.0001 | 3.02 (2.55–3.56) | <0.0001 |
|  | ≥ 85 | 94.9% | 7.24 (6.15–8.53) | | <0.0001 | 6.27 (5.28–7.46) | <0.0001 |
| CCI | |  |  | | <0.0001 |  | <0.0001 |
|  | 0 | 45.1% | Reference | |  | Reference |  |
|  | 1 | 63.9% | 1.80 (1.65–1.96) | | <0.0001 | 1.56 (1.43–1.70) | <0.0001 |
|  | 2 | 73.4% | 2.44 (2.23–2.68) | | <0.0001 | 1.98 (1.80–2.18) | <0.0001 |
|  | 3 | 80.8% | 2.99 (2.68–3.34) | | <0.0001 | 2.25 (2.01–2.52) | <0.0001 |
|  | ≥ 4 | 88.6% | 4.29 (3.85–4.79) | | <0.0001 | 3.23 (2.87–3.62) | <0.0001 |
| Alcohol abuse | |  |  | |  |  |  |
|  | No | 61.2% | Reference | |  | Reference |  |
|  | Yes | 50.9% | 0.82 (0.73–0.92) | | 0.0008 | 1.39 (1.22–1.57) | <0.0001 |
| Atrial fibrillation | |  |  | |  |  |  |
|  | No | 55.7% | Reference | |  | Reference |  |
|  | Yes | 79.3% | 1.91 (1.78–2.05) | | <0.0001 | 1.22 (1.13–1.31) | <0.0001 |
| Coagulopathy | |  |  | |  |  |  |
|  | No | 60.1% | Reference | |  | Reference |  |
|  | Yes | 66.4% | 1.33 (0.97–1.82) | | 0.074 | 1.16 (0.85–1.59) | 0.355 |
| Hypertension | |  |  | |  |  |  |
|  | No | 56.7% | Reference | |  | Reference |  |
|  | Yes | 70.2% | 1.44 (1.35–1.54) | | <0.0001 | 0.95 (0.89–1.02) | 0.189 |

**Supplementary table**. Features associated with 10–year case–fatality after index surgery for cSDH. Results of univariable and multivariable analyses

CCI, Charlson co–morbidity index score; HR, hazard ratio; CI, confidence interval
