# Supplementary figures and images for: Prognosis of patients with operated chronic subdural hematoma
Source: Sci Rep. 2022 Apr 29;12:7020. doi: 10.1038/s41598-022-10992-5 (PMC9054845; doi:10.1038/s41598-022-10992-5)

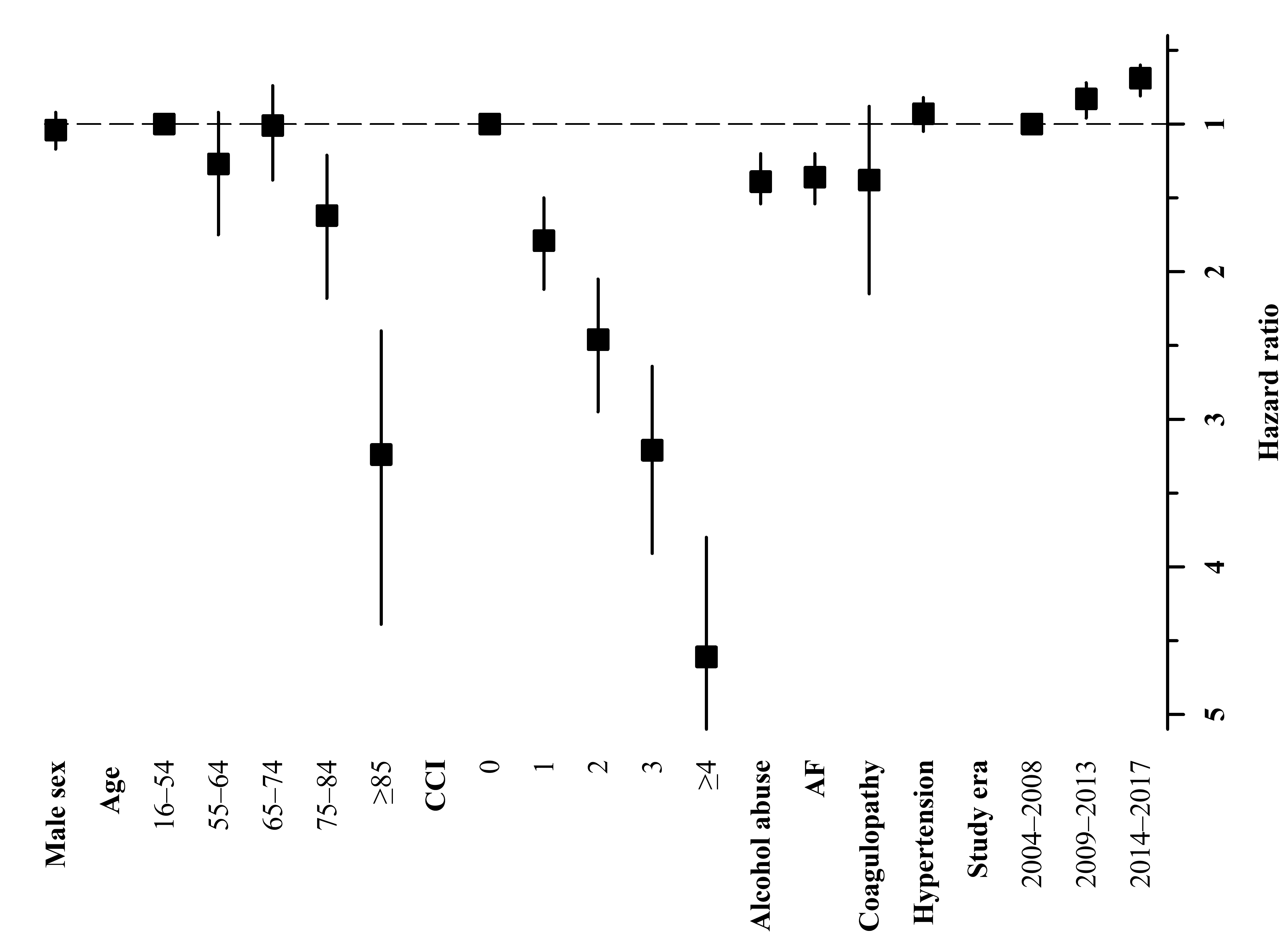

Supplement: Supplementary file 2 — Supplementary Information 2. [file 41598_2022_10992_MOESM2_ESM.tif]

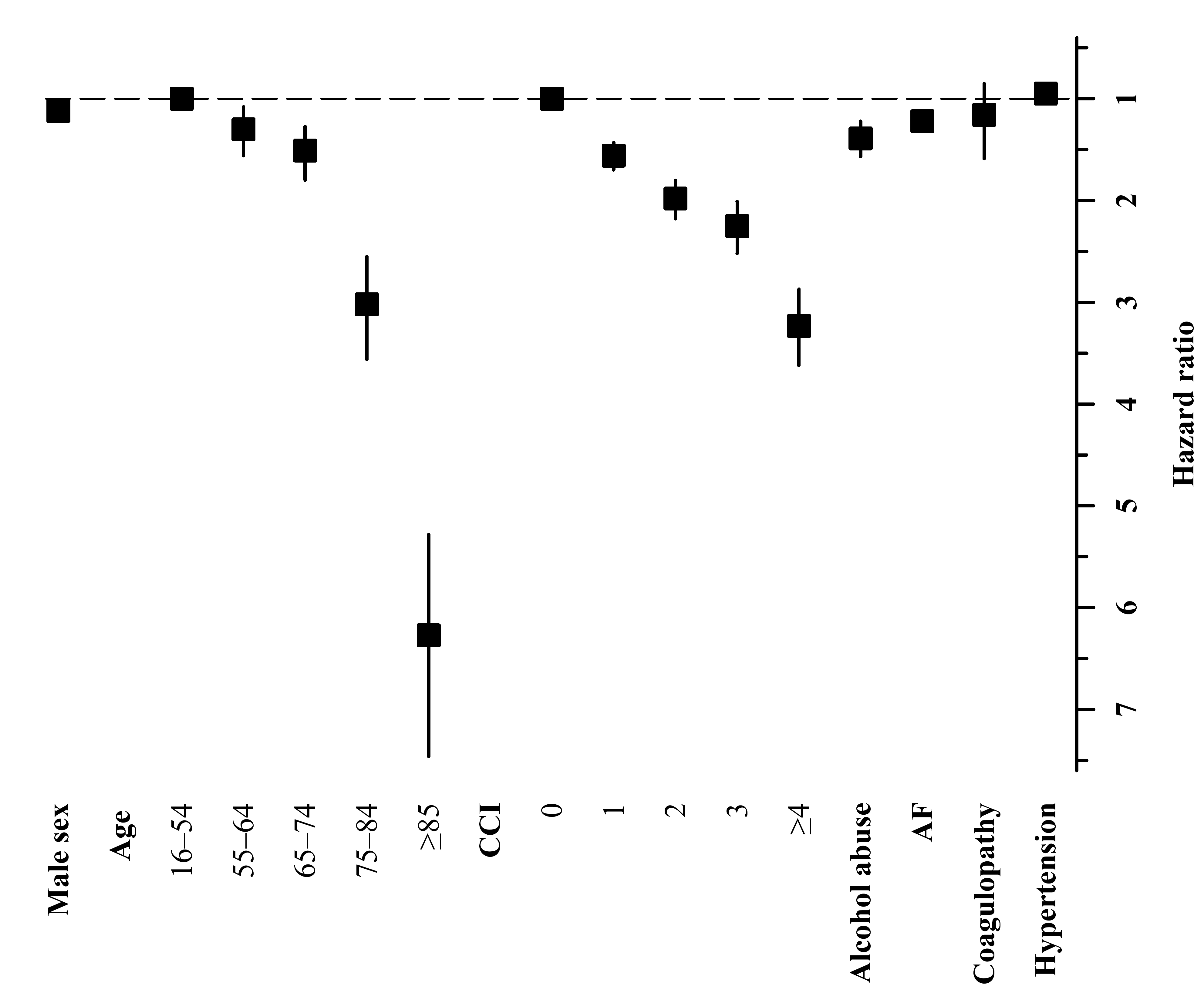

Supplement: Supplementary file 3 — Supplementary Information 3. [file 41598_2022_10992_MOESM3_ESM.tif]

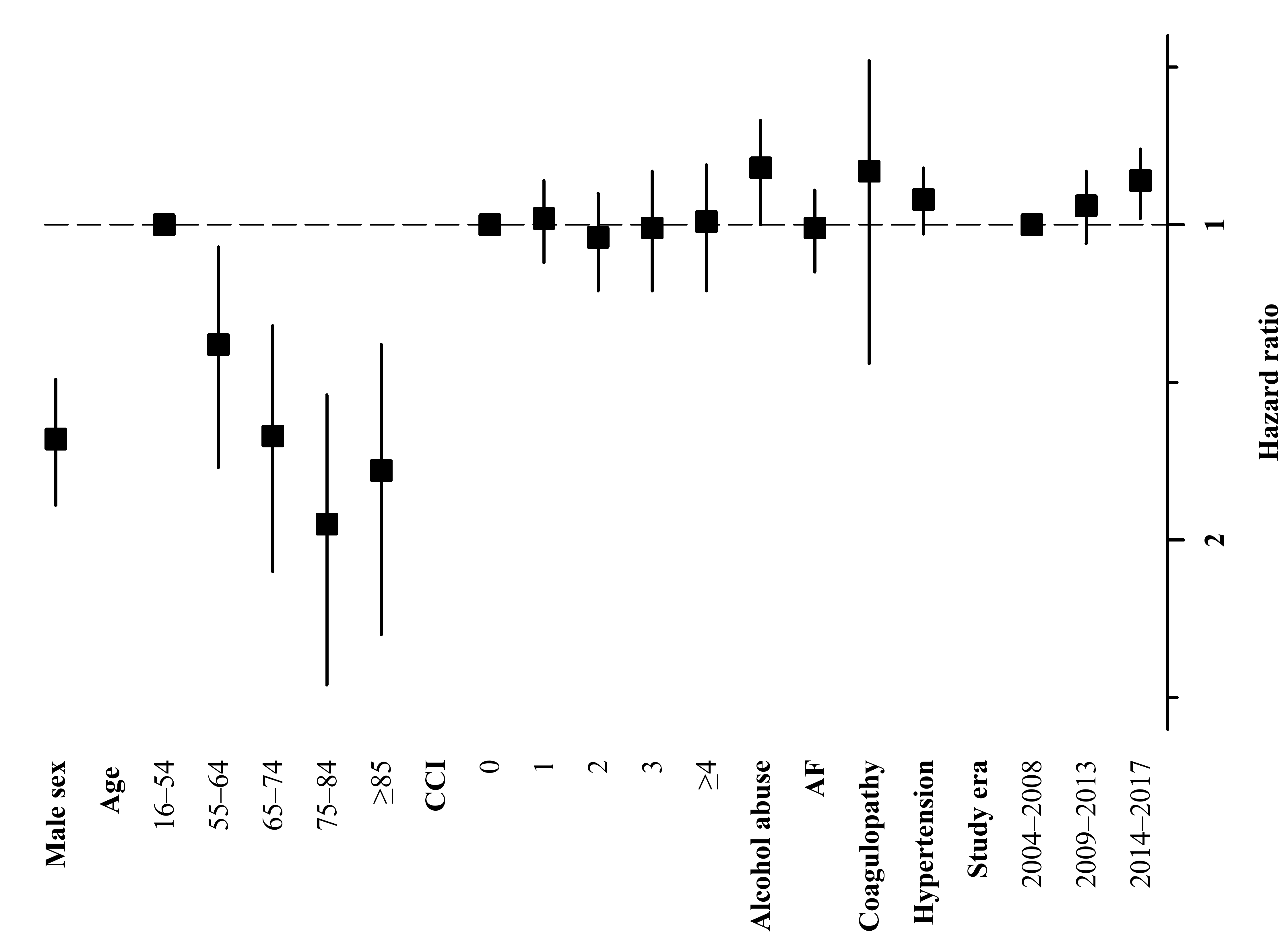

Supplement: Supplementary file 4 — Supplementary Information 4. [file 41598_2022_10992_MOESM4_ESM.tif]
