# Supplementary material for: Prognosis of patients with operated chronic subdural hematoma
Source: Sci Rep. 2022 Apr 29;12:7020. doi: 10.1038/s41598-022-10992-5 (PMC9054845; doi:10.1038/s41598-022-10992-5)
Supplement: Supplementary file 5 — Supplementary Information 5. [file 41598_2022_10992_MOESM5_ESM.docx]

**Supplementary Figure Legends**

**Supplementary figure 1.** Forrest plot of multivariable regression analysis studying features associated with 1–year case–fatality after index surgery for cSDH. For exact values, please refer to Table 3. AF=Atrial fibrillation.

**Supplementary figure 2.** Forrest plot of multivariable regression analysis studying features associated with 10–year case–fatality after index surgery for cSDH. For exact values, please refer to Supplementary Table. AF=Atrial fibrillation.

**Supplementary figure 3.** Forrest plot of multivariable regression analysis studying features associated with re–operation within one year after index surgery for cSDH. For exact values, please refer to Table 4. AF=Atrial fibrillation.
